# Factors Influencing Patient Satisfaction Within the Multistage Online Consultation Process in Internet Hospitals in Beijing: Empirical Analysis

**DOI:** 10.2196/79213

**Published:** 2026-03-16

**Authors:** Yi Wang, Yujia Han, Mengyu Duan, Yani Wang

**Affiliations:** 1 School of Public Health Capital Medical University Beijing China; 2 Yanjing Medical College Capital Medical University Beijing China; 3 Graduate School Chinese Academy of Medical Sciences & Peking Union Medical College Beijing China; 4 Medical Technology College Capital Medical University Beijing China

**Keywords:** internet hospital, multistage of online consultation process, patient satisfaction, SERVQUAL model, service quality, structural equation modeling

## Abstract

**Background:**

Internet hospitals are playing a significant role in medical care with their potential to provide widely accessible outpatient service delivery via information technologies. Current research on patients’ satisfaction with internet hospitals mainly focus on physician-patient relationship and patient demand, and less is considered about the whole process of online consultation.

**Objective:**

This study aims to identity the factors influencing patient satisfaction considering the entire process of online consultation to help physicians deliver better online medical services and to help physical hospitals operate internet hospitals more effectively.

**Methods:**

Based on the service quality (SERVQUAL) theoretical model, questionnaire items were designed for the 5 dimensions of reliability, assurance, responsiveness, empathy, and tangibility. The research samples for data collection were 355 patients on the internet hospital platform operated by a tertiary general hospital in Beijing. Confirmatory factor analysis was performed for dimensions of the measurement model, and path analysis was performed for the structural model.

**Results:**

The current consultation process of internet hospitals, as perceived by patients, did not yet meet their expectations, and the overall satisfaction rate of patients was only 7.15. The 5 dimensions of reliability, responsiveness, empathy, tangibility, and assurance had different positive predictive effects on patient satisfaction in internet hospitals to a certain extent. Among them, tangibility exerted the greatest impact on patient satisfaction in internet hospitals (β=0.25) but obtained the lowest score among the sampled participants (mean=6.78).

**Conclusions:**

Internet hospitals and physicians should focus on the factors of the above dimensions, especially the tangibility of medical services in the multistage online consultation, thus better serving patients and promoting the sustainable development of internet health care.

## Introduction

The internet hospital is a new medical model that realizes online disease diagnosis and treatment services by using information technology to provide services to patients with common diseases or chronic diseases or living in remote and rural areas [1,2]. On such platforms, patients can consult physicians about their disease diagnosis, treatment plan, and prognosis without the limits of time and space [3,4]. In June 2024, the number of internet medical users in China had reached 418 million, accounting for 37.7% of the total internet users. There are 3340 internet hospitals in China, and the annual volume of internet-based medical services provided exceeds 100 million medical visits. The convenience, accessibility, and continuity of medical services in internet hospitals have injected great momentum into the development of China's public health services [5,6].

Internet hospitals have developed rapidly and become an important part of China's medical service system [7]. Many entity hospitals have set up their own internet hospital to provide web-based medical services. Compared with physical hospitals, internet hospitals can reduce patients’ waiting time, increase doctor-patient interaction efficiency, and improve the accessibility of medical service quality [8]. Patient satisfaction is an important basis for evaluating health care quality, and patients will have high satisfaction when they are treated in an expected way and receive high service quality [9]. However, despite the convenience and accessibility of online health care services, there is still room for improvement in patient satisfaction with internet hospitals [3].

Existing research on patients’ satisfaction primarily focus on online physician-patient interaction [4,10] and physician provision [7,11] and tends to examine offline physical hospitals [12,13]. For example, Li et al [4] evaluated the impact of internet hospitals on physician-patient relationship during outpatient visits and suggested improving physicians’ online communication skills and strengthening the level of trust between physicians and patients from a patient perspective. Chang et al [10] investigated how physician-patient online interaction affects the integration of online and offline health services. Wu et al [7] studied patient satisfaction from the perspective of physicians and proposed that physicians’ response, detailed style, and emotional comfort positively affects patient satisfaction. Yang et al [11] explored the effect of physicians’ appearance, smile, and positive emotions on patients’ selection and evaluation behavior and reported interesting and valuable conclusions. However, these studies have only focused on patient satisfaction at a specific stage, without exploring the whole process of patient diagnosis and treatment in internet hospitals.

Online consultation in internet hospitals is a complex and dynamic process, which includes preconsultation, consultation, postconsultation, and online and offline integration [14]. When patients have diagnosis and treatment needs, they can apply to internet hospitals for online consultation services. In the preconsultation stage, the patients need to decide which physician to choose by comparing information displayed in the internet hospitals [15]. Then, patients will communicate with the selected physician to conduct diagnosis and treatment in the consultation stage. In the postconsultation stage, medicines purchased online will be delivered on time without damage. Online and offline integration needs to be performed when the patient's condition is unusual or serious [10].

Patient satisfaction is often linked with health care service quality. Parasuraman et al [16] initially developed a service quality model to explain the potential causes of service quality issues and help managers improve service quality. The model was further improved as a measurement tool, the service quality (SERVQUAL) questionnaire, which consists of 5 dimensions: reliability, assurance, responsiveness, empathy, and tangibility, to measure users' expectation and perception of the service quality provided by the entity and reflects their attitude toward the service provider [17]. Previous studies have evaluated the quality of medical services in hospitals based on the SERVQUAL questionnaire [18-20]. Han et al [3] evaluated patients’ expected and perceived service qualities of internet hospitals by using the service quality questionnaire with a national representative sample. In this study, we designed measure items of the questionnaire based on the 5 dimensions of the SERVQUAL theoretical model to evaluate the service quality from a multistage online consultation process such as preconsultation, consultation, postconsultation, and online-offline integration. The measurement dimensions in each phase of the diagnosis and treatment process can correspond to the SERVQUAL model.

This study aims to identify and clarify the factors influencing patient satisfaction in internet hospitals in Beijing across the multistage online consultation process. Exploring this problem can help such platforms optimize service design, thereby guiding internet hospitals to better serve patients and attract more users. We expect that problems existing in the online diagnosis and treatment process of internet hospitals can be uncovered from the perspective of patients, and suggestions on upgrading and improving internet hospitals can be put forward. Ultimately, internet diagnosis and treatment can better meet the needs of patients in the process of use and management, improve patients' satisfaction with internet hospitals, and thus promote the development of internet diagnosis and treatment.

## Methods

We conducted a web-based cross-sectional survey of patients from an internet hospital platform of Beijing Chaoyang hospital, a tertiary general hospital which provides an integration of online and offline medical services for patients.

### Ethical Considerations

According to the "Ethical Review Measures for Human Life Sciences and Medical Research" (approved by the National Science and Technology Ethics Committee) issued by the National Health Commission and the Ministry of Science and Technology of the People's Republic of China on February 27, 2023 (Document No. GWNJ-KE-2023-4), and according to Article 32 of Chapter 3 of the Ethical Review Regulations [21], this study was exempted from ethic review.

Before the questionnaire survey, the professional staff from the information center of the Beijing Chaoyang hospital carefully verified the questionnaire dimensions to ensure compliance with ethical standards. The survey data were anonymized and kept confidential for the respondents. During the data collection stage, our data was collected through the form of online questionnaires. All participants signed the “Informed Consent Form”, clearly informing them of the research purpose, confidentiality terms, and the right to withdraw voluntarily. In the questionnaire we clearly stated that the collected data would only be used for academic research. We strictly abide by the Personal Information Protection Law. The questionnaires were anonymous and did not involve patients’ names. Consequently, this study did not require ethical approval. Participants who completed a questionnaire could receive a reward of $0.80 USD. During the data processing process, we strictly abided by data protection regulations and took measures to ensure data security.

### Instrument Development

Combining the existing scale of patient satisfaction in offline hospitals and the operating status of internet hospitals in Beijing, questionnaire items were designed based on the 5 dimensions of the SERVQUAL model. Our questionnaire consisted of 2 parts: basic information and satisfaction evaluation. The basic information included characteristics of the patients such as gender, age, education, monthly income, occupation, and the frequency of using internet hospitals. Satisfaction evaluation covered all stages of the patient's internet online diagnosis and treatment process, including prediagnosis, diagnosis, postdiagnosis, payment, purchase of medication, and online and offline referrals, with 4 items on reliability, 5 items on guarantees and responsiveness, 4 items on empathy, 3 items on tangibility, and 4 items on overall patient satisfaction, totaling 25 questions. Multimedia Appendix 1 lists the 25 items used in this study. The items were measured using a 10-point Likert scale (1=strongly disagree, 2=disagree, 3=slightly disagree, 4=somewhat disagree, 5=indifferent, 6=general, 7=somewhat agree, 8=slightly agree, 9=agree, 10=strongly agree).

### Data Collection

To ensure the scientific aspect and rationality of the questionnaire scale, we adopted snowball sampling to conduct a small-scale pretest for patients who have used the internet hospital of a tertiary hospital in Beijing; a total of 44 questionnaires were issued, and 30 valid questionnaires were obtained after eliminating invalid questionnaires, with an effective rate of 68%. The sample data were tested for reliability and validity using SPSS software (version 24.0; IBM Corp). The Cronbach α coefficients of each dimension were greater than 0.7, and the overall Cronbach α coefficient was greater than 0.9, indicating that the internal consistency of the items was better and the overall reliability of the sample was higher. The Kaiser-Meyer-Olkin value was 0.75 and the *P* value was less than .001, meaning that the validity of the sample was acceptable. Therefore, this questionnaire can be used in subsequent studies.

In the formal survey, the questionnaire was released to the internet hospital platform of a tertiary hospital in Beijing, and convenient random sampling was adopted to distribute the questionnaires. To ensure data quality, we implemented a rigorous data screening procedure. Given that it takes 86 seconds at least to read the questionnaire from the beginning to the end, 20 questionnaires with the answer time of less than 86 seconds were excluded. Additionally, 23 questionnaires that selected one option for all questions and 6 questionnaires that failed an attention check were excluded. Finally, 355 valid questionnaires were obtained, with an effective rate of 87.9% (355/404).

### Data Analysis

SPSS (version 27.0) and AMOS (version 23.0; IBM Corp) software were used for data analysis. We first employed descriptive statistics to analyze the demographic information of the patients and descriptive statistics of 25 observed variables. Structural equation modeling was then employed to examine the relationships between measurement items and structural variables to identify the causal relationships between structural variables [22,23]. Among them, confirmatory factor analysis was performed to ensure the reliability and validity for the measurement model, and path analysis was performed to estimate the influencing relationships for the structural model [24].

## Results

### Descriptive Statistics

Table 1 presents the demographic characteristics of the respondents. Among the respondents, males and females were evenly divided, with slightly more females than males. The largest number of people were in the 26-45 years age group, accounting for 31.5% (112/355) of the total sample. Respondents below the high school/secondary school level accounted for nearly half (174/355) of the total sample. Respondents with a monthly income of less than US $409.80 accounted for 38% (135/355) of the total sample. The frequency of using internet hospitals is measured as often (more than 3 times a month), frequently (2-3 times a month), occasionally (once a month), rarely (almost never). More than half (295/355, 83.1%) of the population employed internet hospitals at an average frequency.

**Table 1:** Demographic statistics of the sample (N=355).

Demographic characteristics, categories		Values, n (%)
**Gender**		
	Female	167 (47.1)
	Male	188 (52.9)
**Age (y)**		
	<18	27 (7.6)
	18-25	51 (14.4)
	26-45	112 (31.5)
	46-60	108 (30.4)
	>60	57 (16.1)
**Education**		
	High school or below	174 (49)
	Junior college	70 (19.7)
	Undergraduate	76 (21.4)
	Postgraduate	35 (10)
**Monthly income**		
	Less than US $409.80	135 (38)
	US $409.90-US $819.70	130 (36.6)
	US $819.80-US $1366.10	58 (16.3)
	US $1366.20-US $2049.20	20 (5.6)
	More than US $2049.20	12 (3.4)
Frequency of using internet hospital (frequently)		295 (83.1)

Table 2 provides a descriptive statistics for these items, including item mean and standard deviation, skewness, kurtosis, minimum, and maximum values. The skewness of each item ranged from –0.10 to –1.03, and the kurtosis ranged from 0.30 to 1.75. Since all values fell within the absolute value of 2, the data of each construct were normally distributed [25]. The mean value of each item was greater than the median 5.5, demonstrating that most patients using internet hospitals had a strong sense of identity with the potential variables set by the research. The 5 dimensions of reliability, assurance, responsiveness, empathy, and tangibility proposed in this paper could be regarded as core variables and the following detailed analysis was performed according to the data. However, the average score values of the 5 dimensions were not very high (only around 7), indicating that patients are not very satisfied with the whole process of diagnosis and treatment in the internet hospitals.

**Table 2:** Descriptive statistics for the items.

Constructs (mean), items			Mean (SD)		Skewness		Kurtosis		Minimum		Maximum
**Reliability (mean=6.98)**											
	a1	6.98 (2.12)		–0.87		0.62		1		10	
	a2	6.94 (2.10)		–0.72		0.47		1		10	
	a3	7.00 (2.20)		–0.97		0.83		1		10	
	a4	7.01 (2.12)		–0.95		0.92		1		10	
**Assurance (mean=6.95)**											
	b1	6.95 (2.10)		–0.79		0.53		1		10	
	b2	6.90 (2.15)		–0.83		0.49		1		10	
	b3	6.92 (2.25)		–1.04		0.88		1		10	
	b4	6.97 (2.19)		–0.97		0.73		1		10	
	b5	6.99 (2.22)		–0.95		0.73		1		10	
**Responsiveness (mean=7.05)**											
	c1	6.97 (1.98)		–0.83		0.80		1		10	
	c2	7.06 (1.87)		–0.77		0.92		1		10	
	c3	7.08 (2.01)		–0.98		1.20		1		10	
	c4	7.12 (1.99)		–0.99		1.28		1		10	
	c5	7.01 (2.09)		–0.10		1.28		1		10	
**Empathy (mean=7.02)**											
	d1	7.09 (2.07)		–0.97		1.11		1		10	
	d2	6.88 (2.07)		–0.94		0.98		1		10	
	d3	7.01 (2.07)		–0.88		0.88		1		10	
	d4	7.11 (2.14)		–0.91		0.78		1		10	
**Tangibility (mean=6.78)**											
	e1	6.77 (2.26)		–0.78		0.30		1		10	
	e2	6.77 (2.16)		–0.91		0.61		1		10	
	e3	6.79 (2.25)		–0.84		0.41		1		10	
**Overall satisfaction (mean=7.15)**											
	f1	7.19 (1.96)		–0.89		1.28		1		10	
	f2	7.07 (1.94)		–0.87		1.38		1		10	
	f3	7.19 (1.95)		–0.93		1.17		1		10	
	f4	7.14 (1.90)		–1.03		1.75		1		10	

### Measurement Model

We tested the internal reliability, convergent validity, and discriminant validity for confirmatory factor analysis to evaluate the fitness between the 5 constructs and their indicators. The values of composite reliability, average variance extracted, Cronbach α, and factor loadings were calculated to assess the internal reliability and convergent validity. As shown in Table 3, the factor loading values were all greater than 0.84, reaching the desired level of estimation. The *P* value of the unstandardized coefficients was less than .01, meaning that the observed variables were significant for the latent variables. The reliability coefficients, composite reliability, and Cronbach α were all above the threshold value of 0.7, and average variance extracted were greater than 0.71, indicating that all reflective constructs exhibited good reliability and convergent validity [26,27].

**Table 3:** Construct reliability and validity analysis.

Items			Factor loading		*P* value		Reliability coefficient		Composite reliability			Cronbach α			Average variance extracted	
**Reliability**										0.93			0.93			0.78
	a1	0.89		<.001		0.80										
	a2	0.87		.01		0.75										
	a3	0.88		.01		0.78										
	a4	0.89		—^a^		0.79										
**Assurance**										0.94			0.94			0.77
	b1	0.87		<.001		0.76										
	b2	0.87		<.001		0.76										
	b3	0.87		<.001		0.76										
	b4	0.89		<.001		0.80										
	b5	0.87		—		0.75										
**Responsiveness**										0.92			0.93			0.71
	c1	0.85		.01		0.71										
	c2	0.84		<.001		0.71										
	c3	0.84		<.001		0.70										
	c4	0.84		<.001		0.70										
	c5	0.86		—		0.74										
**Empathy**										0.91			0.92			0.73
	d1	0.85		<.001		0.73										
	d2	0.86		.01		0.73										
	d3	0.84		.01		0.71										
	d4	0.87		—		0.75										
**Tangibility**										0.90			0.90			0.75
	e1	0.89		<.001		0.80										
	e2	0.86		<.001		0.74										
	e3	0.86		—		0.74										
**Overall satisfaction**										0.91			0.91			0.71
	f1	0.85		—		0.72										
	f2	0.84		<.001		0.71										
	f3	0.84		.01		0.70										
	f4	0.85		<.001		0.72										

^a^Not applicable.

For discriminant validity, the square root of average variance extracted for each construct on the diagonal was much larger than the correlation coefficients of the other constructs, indicating good discriminant validity of the measurement model, as shown in Table 4. The correlation coefficients of assurance, reliability, responsiveness, empathy, tangibility, and overall patient satisfaction were 0.88, 0.88, 0.84, 0.85, 0.87, and 0.51, respectively, with *P* values of less than .001, indicating that there was a significant positive correlation between the constructs and patient satisfaction.

**Table 4:** Discriminant validity analysis.

	Reliability	Assurance	Responsiveness	Empathy	Tangibility	Overall satisfaction
Reliability	0.88	—^a^	—	—	—	—
Assurance	0.36	0.88	—	—	—	—
Responsiveness	0.34	0.48	0.84	—	—	—
Empathy	0.36	0.42	0.39	0.85	—	—
Tangibility	0.35	0.40	0.33	0.35	0.87	—
Overall satisfaction	0.36	0.42	0.39	0.39	0.43	0.51

^a^Not applicable.

### Structural Model

We performed absolute fit, added fit, and reduced fit to verify the fit degree of the structural model. The fitting effect of the structural model is shown in Table 5, with a chi-square of 321.6, a df value of 260, and chi-square minimum/df of 1.24, which was between 1 and 3. The goodness-of-fit index, measuring how much of the variance and covariance of the observed data can be explained by the model, was 0.93 greater than the threshold of 0.90 and the root mean square error of approximation value, which measures the gap between the theoretical model and the perfect fitting model, was 0.03 less than the threshold of 0.05, indicating that the model had a good absolute fit. The normed fit index value, incremental fit index value, Tucker-Lewis Index, and comparative fit index explain the degree of fit of the hypothetical model and the independent model from various aspects, whose values were all greater than the threshold of 0.90, meaning that the model had a good added fit with the actual data. The Parsimony-adjusted normed fit index value, Parsimony-adjusted comparative fit index value, and Parsimony-adjusted goodness-of-fit index explain the simplicity of the model based on the assessment of fit. The values were all greater than the threshold of 0.5, indicating that the model had a reduced fit [28]. Overall, the fitness of the structural model was good.

**Table 5:** Goodness-of-fit test.

Indicators		Model indicator values	Reference standard	Whether or not fit
**Absolute fit**				
	Chi-square minimum/df	1.24	<3	Fit
	Root mean square error of approximation	0.03	<0.05	Fit
	Goodness-of-fit index	0.93	>0.9	Fit
**Added fit**				
	Normed fit index	0.96	>0.9	Fit
	Incremental fit index	0.99	>0.9	Fit
	Tucker-Lewis index	0.99	>0.9	Fit
	Comparative fit index	0.99	>0.9	Fit
**Reduced fit**				
	Parsimony-adjusted normed fit index	0.83	>0.5	Fit
	Parsimony-adjusted comparative fit index	0.86	>0.5	Fit
	Parsimony-adjusted goodness-of-fit index	0.75	>0.5	Fit

The results of structural model are reported in Table 6, which presents the estimated coefficients, the standardized and nonstandardized coefficients, standard error, critical ratio, and the associated *P* values. The coefficients represented the factor loading from patient satisfaction to the 5 dimensions of reliability, assurance, responsiveness, empathy, and tangibility. Standard error was used to judge the relationship between different variables in the model, as well as its strength and direction, the smaller and better the standard error, indicating that the parameter estimation was more reliable, and vice versa, indicating that the parameter estimation of the uncertainty was greater. Critical ratio was used to judge whether the variables in the model had a good continuity. If it was greater than or equal to 1, it showed that the model had good continuity and could be considered existing important and stable relationships between the variables. On the contrary, it meant that the model had a large uncertainty and need to be further modified. A *P* value of less than .05 indicated that the dimension had a positive effect on patient satisfaction.

**Table 6:** Path coefficients and significance level analysis.

	Standardized coefficient	Nonstandardized coefficient	Standard error	Critical ratio	*P* value
Reliability	0.15	0.13	0.05	2.39	.02
Assurance	0.12	0.11	0.05	2.10	.04
Responsiveness	0.15	0.14	0.06	2.56	.01
Empathy	0.15	0.13	0.05	2.50	.01
Tangibility	0.25	0.22	0.05	4.28	.01

As shown in Table 6, the standardized coefficients of the 5 dimensions of assurance, reliability, responsiveness, empathy, and tangibility on patient satisfaction were all greater than 0, and the *P* value was less than .05, which proved that there was a significantly positive correlation between all 5 dimensions on the overall satisfaction of patients in the internet hospitals, that is, the improvement of the 5 dimensions could correspondingly affect the improvement of overall patient satisfaction. The standard errors were all positive and small in value, indicating that there was a positive effect of the 5 dimensions on overall satisfaction, and the parameter estimation of the path was reliable. The critical values were all greater than 1, indicating that the model had good continuity and there was an important and significant relationship between the 5 dimensions and overall patient satisfaction. In summary, it could be seen that the 5 dimensions played a positive role in predicting overall patient satisfaction.

## Discussion

### Main Findings and Suggestions

This study aims to investigate the factors influencing patient satisfaction in the multistage online consultation process based on 5 dimensions of the SERVQUAL theoretical model. To summarize, the current consultation process of internet hospitals as perceived by patients did not yet meet their expectations, and the overall satisfaction rate of patients was only 7.15. We will discuss this in detail from the following aspects.

First, our study reveals a critical paradox: among the various dimensions measuring the service quality of internet hospitals, tangibility received the lowest patient ratings, but it was identified as the most influential factor affecting overall satisfaction. The results are similar to other studies using the SERVQUAL model that emphasized the importance of tangibility [19,20]. The intangible nature of the service remains a primary bottleneck for the development of internet health care, while successfully making the virtual service tangible can yield significant returns in terms of patient trust and satisfaction. However, improving tangibility necessitates a profound experience redesign. The online user interface should evolve beyond basic functionality to become a communication medium itself, which can be achieved through visual service flow maps and multidimensional presentations of the medical teams, thereby making the intangible diagnostic and treatment process clear, predictable, and structured [10]. Besides, hospitals should integrate online and offline support systems to create a seamless chain of trust by establishing end-to-end tracking systems for prescription approval, medication delivery, and offline examination appointments. In summary, the future development of internet hospitals should not solely focus on stacking technological features but must strive to transform cold online interactions into a warm and perceptible service experience. By implementing the proposed 2-pronged strategy for enhancing tangibility, internet hospitals can effectively mitigate their inherent disadvantage of service intangibility, which will pave the way for building robust patient satisfaction and achieving sustainable growth.

Second, the findings clearly show that reliability is a core factor driving patient satisfaction in internet hospitals. Reliability assesses the ability of internet hospitals to deliver services accurately and dependably [4,18]. To strengthen reliability, internet hospitals must strive to consistently fulfill their promises to patients throughout the entire process from preconsultation, during consultation, to postconsultation. First, preconsultation focuses on establishing clear expectations, requiring physicians to adhere strictly to appointment times and ensuring complete fee transparency through detailed cost breakdowns. Second, during and postconsultation, the emphasis shifts to delivering a high-quality service loop. Previous studies have shown that online physicians are a key factor in improving patient satisfaction [11]; internet hospitals should be aware of the importance of physicians’ appearance and smile on patients’ selection and evaluation behavior. Physicians should communicate with professionalism and empathy (eg, a smile) to build trust and compensate for the lack of face-to-face interaction. Concurrently, drug delivery must be punctual, accurate, and with intact packaging, representing the final fulfillment of the service promise. Finally, underpinning all services is technical assurance. Internet hospitals must prioritize network security and patient privacy protection, maintained by dedicated IT teams to ensure system stability and data security [8]. In essence, by standardizing processes, ensuring transparency, and rigorously honoring commitments within this framework, internet hospitals can systematically build reliability, leading to a significant increase in patient satisfaction.

Responsiveness also demonstrated a significant positive impact on patient satisfaction in internet hospitals. Notably, among the 5 SERVQUAL dimensions, responsiveness received the highest score from the participants. This dimension pertains to the service efficiency of internet hospitals, reflected specifically in the promptness of staff replies to patient inquiries and whether the exact time for each service is communicated in advance [29]. To continuously improve responsiveness, internet hospitals need to optimize multiple aspects. First, IT support should be strengthened to ensure that medical staff can promptly address patient needs during consultations and provide quick feedback and solutions to postconsultation issues. Second, in response to patient-reported problems such as difficulty contacting doctors and delayed responses, root causes such as network latency, system lag, or unreasonable interface design should be thoroughly analyzed and addressed through technological upgrades and process optimization. Additionally, internet hospitals should focus on enhancing service synergy, reducing communication delays, and avoiding consultation interruptions or efficiency losses due to poor communication. On the other hand, internet hospitals should further expand the application scenarios of responsiveness. For example, timely push of hospital resource information (eg, doctor schedules, latest services) and the implementation of rapid response mechanisms for payment, refunds, and other processes can comprehensively improve service efficiency and patient experience. In the future, internet hospitals can leverage intelligent technologies (eg, AI pre-replies, smart triage) to enhance responsiveness, reducing the workload of medical staff while further improving service efficiency and patient satisfaction.

Fourth, a significant positive correlation exists between empathy and patient satisfaction in internet hospitals. Improving the empathy of internet hospitals needs to focus on whether patients feel cared for in the whole process of diagnosis and treatment [30]. When internet hospitals provide personalized services, demonstrate genuine concern, and proactively address patient needs, patients feel valued, which fosters positive emotions and, consequently, increases their satisfaction [3]. For instance, internet hospitals should cater to the needs of special patient groups such as older adults and persons with disabilities. Implementing dedicated application modes such as an “Elderly Mode” with optimized font styles and sizes can directly address their specific requirements and reflect a commitment to inclusive, humanistic care. Furthermore, as internet hospitals primarily serve patients with chronic conditions or those facing mobility challenges, the scheduling system must be patient-centric. The allocation of appointment slots and the number of available time slots should not only align with patient demand but also be balanced reasonably with physicians' available consultation hours. Additionally, offline hospitals can enhance empathy by establishing dedicated service counters for their internet hospital services. These counters could assist patients with practical tasks such as printing electronic prescriptions or reports and serve as an introduction point for first-time users, guiding them through the platform's features. This integration of online and offline support embodies a holistic and empathetic approach to patient care.

Finally, among the 5 service quality dimensions, assurance was found to have the least impact on patient satisfaction, a finding that aligns with practical realities. In the context of this study, assurance primarily pertains to the technical competence of online physicians and the effectiveness of the treatment received. Patients who generally lack professional medical knowledge and face information asymmetry are often unable to directly evaluate this dimension, making the sense of assurance less tangible in an internet hospital setting. Nonetheless, it is indisputable that physicians' professional knowledge remains the cornerstone of a credible internet hospital service [14]. Therefore, entities operating these platforms, including traditional hospitals, must prioritize the rigorous selection and training of online physicians. Deploying high-caliber medical talent is essential to enhance the perceived assurance and, consequently, patient satisfaction. To this end, offline hospitals should assign their most qualified medical teams to online duties, ensuring that the quality of consultation achieves parity with in-person visits. Furthermore, strengthening the integration between online and offline services is crucial. By creating seamless referral channels and strategically shifting appropriate follow-up care burdens from offline to online platforms, health care providers can build a cohesive and trustworthy system. This integration not only enhances assurance but also encourages patient adoption and sustained use of internet hospitals throughout their entire health care journey.

### Conclusion and Limitations

This study analyzes patient satisfaction in the multistage online consultation process in internet hospitals. Based on the SERVQUAL model, we designed questionnaire questions for the 5 dimensions of reliability, assurance, responsiveness, empathy, and tangibility, and collected data from patients of the internet hospital of a tertiary hospital in Beijing. We found that the 5 factors of reliability, responsiveness, empathy, tangibility, and assurance all had different positive predictive effects on patient satisfaction in internet hospitals to a certain extent. Our results provide patients, physicians, and online health communities with guidelines that support them in widely adopting internet hospitals and effectively improving their decision-making.

This paper has some shortcomings while achieving certain results. First, this paper determined 25 observed variables of the 5 dimensions according to previous literature and the actual situation of a tertiary hospital internet hospital in Beijing. However, the operation mode of each internet hospital is different, and the applicability of the variable selection is worth considering. Second, the cross-sectional design limits the ability to establish causal relationships between the 5 dimensions of SERVQUAL theoretical model and patient satisfaction. Future research could use longitudinal studies to examine the pathways of causality and the possible changes in patient satisfaction over time. In addition, the survey respondents were patients using online health services in Beijing. As an emerging thing, patients in different regions may present different satisfaction levels due to ethnic and cultural differences, and future research could explore patients in different regions.

## Appendix

Multimedia Appendix 1 Measurement items of constructs.

## Abbreviations

SERVQUAL: service quality
