# Supplementary material for: Factors Influencing Patient Satisfaction Within the Multistage Online Consultation Process in Internet Hospitals in Beijing: Empirical Analysis
Source: JMIR Hum Factors. 2026 Mar 16;13:e79213. doi: 10.2196/79213 (PMC13036401; doi:10.2196/79213)
Supplement: Multimedia Appendix 1 [file humanfactors_v13i1e79213_app1.docx]

**Multimedia Appendix 1.** Measurement items of constructs.

| Constructs | items | Medical consultation process | Measures |
| --- | --- | --- | --- |
| Reliability | a1 | During diagnosis | Online diagnosis and treatment doctors have high visibility, high professional and technical level, and have certain authority. |
|  | a2 | During diagnosis | The effect of online diagnosis and treatment is good, and it can achieve similar curative effect with offline diagnosis and treatment. |
|  | a3 | During diagnosis | Online diagnosis and treatment process can see accurate information and medical records. |
|  | a4 | Transfer treatment | The online number can be booked during the offline diagnosis and treatment period for long-term follow-up visits, and the offline number can be booked during the online diagnosis and treatment period for complex medical conditions. |
| Assurance | b1 | Before diagnosis | When seeing a doctor online, the doctor can follow the appointment time to enter the chat interface. |
|  | b2 | During diagnosis | Online doctors have high integrity, good character and are worthy of patients' trust. |
|  | b3 | After diagnosis | After purchasing medicine online, the medicine can be accurately delivered according to the agreed time and without damage. |
|  | b4 | Whole procedure | Online diagnosis and treatment fee details are clear and reasonable, in line with national norms, and consistent with offline. |
|  | b5 | Whole procedure | The Internet hospital fully encrypts patient information to protect patient privacy. |
| Responsiveness | c1 | During diagnosis | In online diagnosis and treatment, doctors can quickly receive treatment, reply to messages in time, and solve problems. |
|  | c2 | Paying | The payment and refund of the Internet hospital are timely, and the payment can be quickly returned when the problem is encountered. |
|  | c3 | Whole procedure | When the use of the Internet hospital treatment encounters unexpected problems, there are special channels to contact the staff, and the staff can reply in a timely manner. |
|  | c4 | After diagnosis | The Internet hospital can deal with patients' complaints in a timely manner, and carry out rectification and improvement. |
|  | c5 | Whole procedure | The Internet hospital can push hospital resources or information in a timely manner, and inform the exact time and precautions of various services. |
| Empathy | d1 | Before diagnosis | Online diagnosis and treatment appointment time and number of sources can meet the needs of patients. |
|  | d2 | Whole procedure | Online service staff can proactively and patiently understand the needs of patients, especially for special patients to give care and help. |
|  | d3 | Whole procedure | Online page fonts, pictures, size, etc. take into account the needs of special groups, make people feel comfortable. |
|  | d4 | Integration of online and offline medical services | Offline hospitals set up Internet hospital Windows to solve patients' problems, such as printing electronic bills, especially to help patients and special groups who use Internet hospitals for the first time. |
| Tangibility | e1 | Client Operating System | The interface design of Internet hospital is simple and beautiful, and the pictures are clear. The online payment program operates smoothly and jumps smoothly. |
|  | e2 | Client Operating System | When using the Internet hospital, you can see a detailed introduction of the online doctors and medical teams, including areas of expertise. |
|  | e3 | Integration of online and offline medical services | The Internet hospital is equipped with complete offline equipment, such as self-service check-in machines, offline medicine cabinets, etc., with clear guidelines and operation guidance. |
| Overall satisfaction | f1 | - | Your overall satisfaction with the Internet hospital. |
|  | f2 | - | To what extent the Internet hospital service meets your expectations. |
|  | f3 | - | You will be willing to use Internet hospital again. |
|  | f4 | - | You would recommend the willingness of others to use the Internet hospital. |
